# Correlation between Pre-Ovulatory Follicle Diameter and Follicular Fluid Metabolome Profiles in Lactating Beef Cows

**DOI:** 10.3390/metabo11090623

**Published:** 2021-09-14

**Authors:** Casey C. Read, Lannett Edwards, Neal Schrick, Justin D. Rhinehart, Rebecca R. Payton, Shawn R. Campagna, Hector F. Castro, Jessica L. Klabnik, Emma J. Horn, Sarah E. Moorey

**Affiliations:** 1 Department of Animal Science, University of Tennessee, Knoxville, TN 37996, USA; cread6@utk.edu (C.C.R.); jedwards@utk.edu (L.E.); fschrick@utk.edu (N.S.); jrhinehart@utk.edu (J.D.R.); rfrazor@utk.edu (R.R.P.); jbradf21@utk.edu (J.L.K.); ehorn5@utk.edu (E.J.H.); 2 Department of Chemistry, University of Tennessee, Knoxville, TN 37996, USA; campagna@utk.edu (S.R.C.); hcastrog@utk.edu (H.F.C.)

**Keywords:** follicular fluid, metabolome, follicle size, induced ovulation, cumulus-oocyte complex, beef cattle

## Abstract

Induced ovulation of small pre-ovulatory follicles reduced pregnancy rates, embryo survival, day seven embryo quality, and successful embryo cleavage in beef cows undergoing fixed-time artificial insemination. RNA-sequencing of oocytes and associated cumulus cells collected from pre-ovulatory follicles 23 h after gonadotropin-releasing hormone (GnRH) administration to induce the pre-ovulatory gonadotropin surge suggested reduced capacity for glucose metabolism in cumulus cells of follicles ≤11.7 mm. We hypothesized that the follicular fluid metabolome influences metabolic capacity of the cumulus-oocyte complex and contributes to reduced embryo cleavage and quality grade observed following induced ovulation of small follicles. Therefore, we performed a study to determine the correlation between pre-ovulatory follicle diameter and follicular fluid metabolome profiles in lactating beef cows (Angus, *n* = 130). We synchronized the development of a pre-ovulatory follicle and collected the follicular contents approximately 20 h after GnRH administration. We then performed ultra-high performance liquid chromatography—high resolution mass spectrometry (UHPLC-HRMS) metabolomic studies on 43 follicular fluid samples and identified 38 metabolites within pre-ovulatory follicles of increasing size. We detected 18 metabolites with a significant, positive correlation to follicle diameter. Individual and pathway enrichment analysis of significantly correlated metabolites suggest that altered glucose and amino acid metabolism likely contribute to reduced developmental competence of oocytes when small pre-ovulatory follicles undergo induced ovulation.

## 1. Introduction

Decreased pre-ovulatory follicle diameter at the time of pharmacological induction of ovulation was associated with lower pregnancy rates and/or reduced embryo survival in beef cattle undergoing fixed-time artificial insemination (FTAI) [1,2,3,4]. Such reductions in fertility were likely the result of poor preparation of the maternal environment for pregnancy establishment and the ovulation of an oocyte with reduced developmental competency [2,5,6,7]. When beef cows were induced to ovulate a pre-ovulatory follicle <12.5 mm (small) versus a follicle ≥12.5 mm (large) in diameter, embryo quality grade and the probability of recovering a cleaved embryo seven days after FTAI were decreased [7]. RNA-sequencing of pools of four oocytes or associated cumulus cells recovered from small (≤11.7 mm) and large (≥12.7 mm) follicles ~23 h after administration of gonadotropin releasing hormone (GnRH) to stimulate the pre-ovulatory gonadotropin surge suggested decreased metabolic capacity of cumulus cells from small follicles [8]. 

Cumulus cells possess high glycolytic activity and are responsible for producing and transporting metabolites such as pyruvate, lactate, NADH, and FADH2 to the oocyte via gap junctions [9]. These metabolites are then utilized as substrates or electron carriers to maintain production of adenosine triphosphate (ATP) via oxidative phosphorylation in the maturing oocyte and pre-blastocyst stage embryo [10,11,12]. The oocyte and early embryo rely on stores of pyruvate, ATP, and other metabolites to support the metabolic requirements of oocyte maturation, fertilization, and sustained development through the blastocyst stage [13,14,15,16]. During the antral stages of folliculogenesis, the follicular fluid provides many of the nutrients, metabolic compounds, and signaling molecules that are essential for the accumulation of stockpiles of metabolic substrates within the oocyte [9,17]. Follicular fluid, which is derived from plasma and the secretions of intrafollicular cells, both influences and is the result of varied metabolic activities within the follicular cells [18,19,20,21,22,23,24]. Therefore, exploration of the follicular fluid milieu provides insight into follicular cell function. The follicular fluid metabolome is dependent on the stage of folliculogenesis, the developmental stage of the oocyte, and stage of the estrous cycle [25,26]. Follicular fluid metabolome profiles have been linked to fertility in multiple species, and multiple follicular fluid components have been identified as biomarkers of oocyte developmental competence [27,28,29]. Though studies of the follicular fluid metabolome have been performed in humans and dairy cows, little focus has been placed on the impact of follicle diameter at induced ovulation on the follicular fluid and resulting cellular functions of the granulosa cells or cumulus-oocyte complex in beef cows. Studies that investigate the relationship between pre-ovulatory follicle size and the follicular fluid milieu are essential to further understanding reduced oocyte developmental competence and subsequent fertility when beef cows are induced to ovulate a small pre-ovulatory follicle. We hypothesized that size-dependent changes in the follicular fluid’s metabolome influence metabolic capacity of the cumulus-oocyte complex that contribute to reduced embryo cleavage and quality grade observed following induced ovulation of small pre-ovulatory follicles. Therefore, we designed a study with the objective to profile the metabolome of follicular fluid collected from pre-ovulatory follicles ~20 h after GnRH administration to induce the pre-ovulatory gonadotropin surge and determine the correlation between pre-ovulatory follicle diameter at GnRH administration and follicular fluid metabolite concentrations. Follicular fluid samples collected at this timepoint represent the intrafollicular conditions present during the final stages of oocyte maturation and should be indicative of critical metabolic processes within the follicle just prior to ovulation. We utilized ultra-high performance liquid chromatography—high resolution mass spectrometry (UHPLC-HRMS) to identify metabolites within pre-ovulatory follicles of increasing size and determined metabolites with a significant correlation to follicle diameter. 

## 2. Results and Discussion

### 2.1. Animal Data

**Figure 1:**
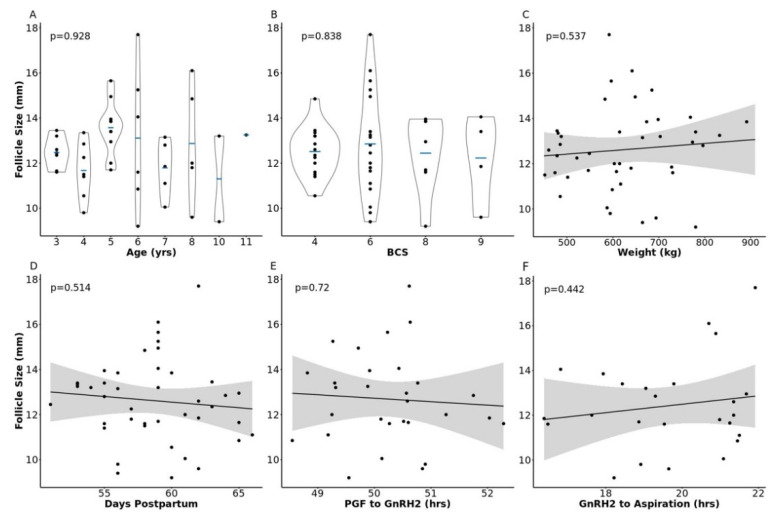
Relationship between pre-ovulatory follicle diameter and cow phenotype or timeline parameters. Panels (**A**–**F**) depict the relationship between follicle size at gonadotropin-releasing hormone administration to induce a pre-ovulatory gonadotropin surge (GnRH2) and cow age, body condition score (BCS), weight, days postpartum, hours from prostaglandin F2α administration (PGF) to GnRH2 administration, and hours from GnRH2 administration to follicle aspiration, respectively.

We utilized follicular fluid that was collected from the pre-ovulatory follicle of 43 lactating beef cows for this study. Follicle size at GnRH administration to induce the pre-ovulatory gonadotropin surge (GnRH2) ranged from 9.2–17.7 mm (12.62 ± 0.28 mm). This measure is consistent with pre-ovulatory follicle diameters observed in multiple studies across varied locations and beef breeds [2,3,7,8,30,31]. To examine the influence of cow phenotype or study timeline on follicle diameter, we utilized analysis of variance or linear regression to quantify relationships between cow descriptive characteristics or study timeline and pre-ovulatory follicle size at GnRH2 administration. There was no relationship between pre-ovulatory follicle diameter at GnRH2 administration and cow age (*p* = 0.93), body condition score (BCS; scale of 1 to 9 in which 1 = emaciated and 9 = obese, *p* = 0.84), weight (*p* = 0.54), days postpartum at aspiration (*p* = 0.51), hours between prostaglandin F2α (PGF) and GnRH2 administration (*p* = 0.72), or hours between GnRH2 administration and pre-ovulatory follicle aspiration (*p* = 0.44; Figure 1). 

Average cow age (5.75 ± 0.34 years) was consistent with previous reports of mean beef cow longevity of approximately four to eight years, depending on production and geographical location [32,33]. Average body condition score (6.023 ± 0.26) and weight (623.3 ± 17.1 kg) of animals included in this study were as expected in beef cattle operations; however, the upper BCS range in our cohort extended to beyond the BCS range commonly observed in beef production scenarios [34]. Cows were 58.7 ± 0.56 days postpartum at the time of follicle aspiration. Though 62.9 ± 2.1 days was reported as the mean postpartum anestrous period of Angus-sired cows [35], all animals in the current study were exposed to a seven day progestin treatment (Eazi-Breed CIDR^®^) during pre-synchronization procedures. The majority (84%) of the animals in this study had a corpus luteum present on the ovaries at the onset of synchronization, and 47% had displayed estrus by 66 h post PGF administration and CIDR removal in the pre-synchronization protocol. Neither parameter influenced pre-ovulatory follicle diameter at GnRH2 administration (*p* = 0.23 and *p* = 0.53, respectively). The time between PGF and GnRH2 administration (50.25 ± 0.17 h) was similar to previous studies conducted by our lab and collaborators that determined the impact of pre-ovulatory follicle diameter on beef cow pregnancy rates and cumulus-oocyte complex transcriptome profiles [2,8]. The time elapsed between GnRH2 administration and follicle aspiration (19.59 ± 0.34 h) allows us to collect samples as close as possible to ovulation and was similar to our previous study that examined the impact of follicle diameter on cumulus-oocyte complex transcriptome profiles [8]. Samples collected at this timepoint represent intrafollicular conditions post gonadotropin surge [36], when somatic follicular cells have begun luteinization, cumulus cells have expanded, and the oocyte’s nuclear maturation has progressed through metaphase one (MI) and has reached or is nearing metaphase two (MII) [37,38,39,40]. The metabolome of follicular fluid collected at this timepoint should reveal critical cellular processes taking place in the granulosa cells and cumulus-oocyte complex during the final stages of oocyte maturation, while highlighting potential processes that are altered when small pre-ovulatory follicles are prematurely exposed to a pharmacologically-induced gonadotropin surge.

### 2.2. Metabolome Profiles of Follicular Fluid Collected from Pre-Ovulatory Follicles of Lactating Beef Cows

**Figure 2:**
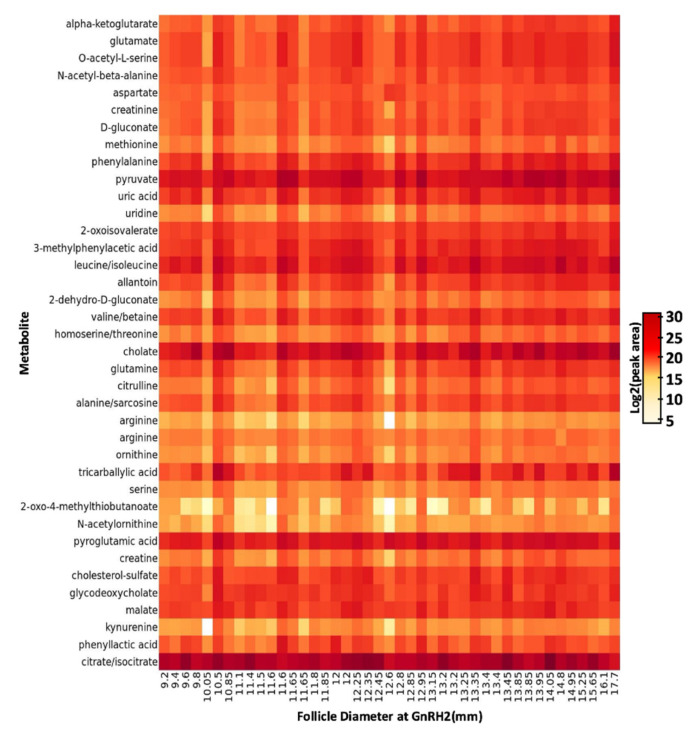
Heatmap of 38 metabolites detected in pre-ovulatory follicular fluid. Metabolites are listed as row titles and the pre-ovulatory follicle diameter at the time of gonadotropin releasing hormone administration to induce a pre-ovulatory gonadotropin surge (GnRH2) corresponding to each follicular fluid sample is listed under each column. Log2 (peak area) of each metabolite within each sample is designated by increasing color intensity. The color intensity scale depicts differences in peak area across all metabolites and demonstrates metabolites present in higher (darker color) and lower (lighter color) concentration in the follicular fluid.

To our knowledge, this is the first study that investigates size-related changes within the follicular fluid metabolome of pre-ovulatory follicles in beef cattle during the peri-ovulatory period of follicular development. The follicular fluid is primarily derived from the circulating plasma and its composition is influenced by both the plasma and products excreted by the intrafollicular cells [24]. The abundance of many metabolic substrates present in the follicular fluid is closely correlated to metabolic activity of the follicular cells [29]. Thirty-eight metabolites were identified in the follicular fluid of bovine pre-ovulatory follicles following UHPLC-HRMS and metabolite identification with metabolic analysis visualization engine (MAVEN; Figure 2). Metabolites identified in the follicular fluid were predominantly amino acids and their derivatives. We also identified a number of glucose derivatives and tricarboxylic acid (TCA) cycle intermediates or derivatives as well as fatty acids, the steroid ester cholesterol sulfate, and the nucleoside uridine. The majority of the metabolites identified in this study have been previously identified in the follicular fluid of cattle, humans, pigs, sheep, and horses [26,41,42,43,44,45,46,47,48,49,50,51,52,53,54,55,56,57,58,59,60,61,62]. To our knowledge, the metabolites N-acetyl-beta-alanine, *O*-acetyl-l-serine, 2-oxo-4-methylthiobutanoate, 3-methylphenylacetic acid, phenyllactic acid, N-acetylornithine, tricarbalylic acid, 2-dehydro-D-gluconate, and D-gluconate are more novel components of the mammalian follicular fluid metabolome. 

### 2.3. The Impact of Increasing Pre-Ovulatory Follicle Diameter on the Follicular Fluid Metabolome

**Figure 3:**
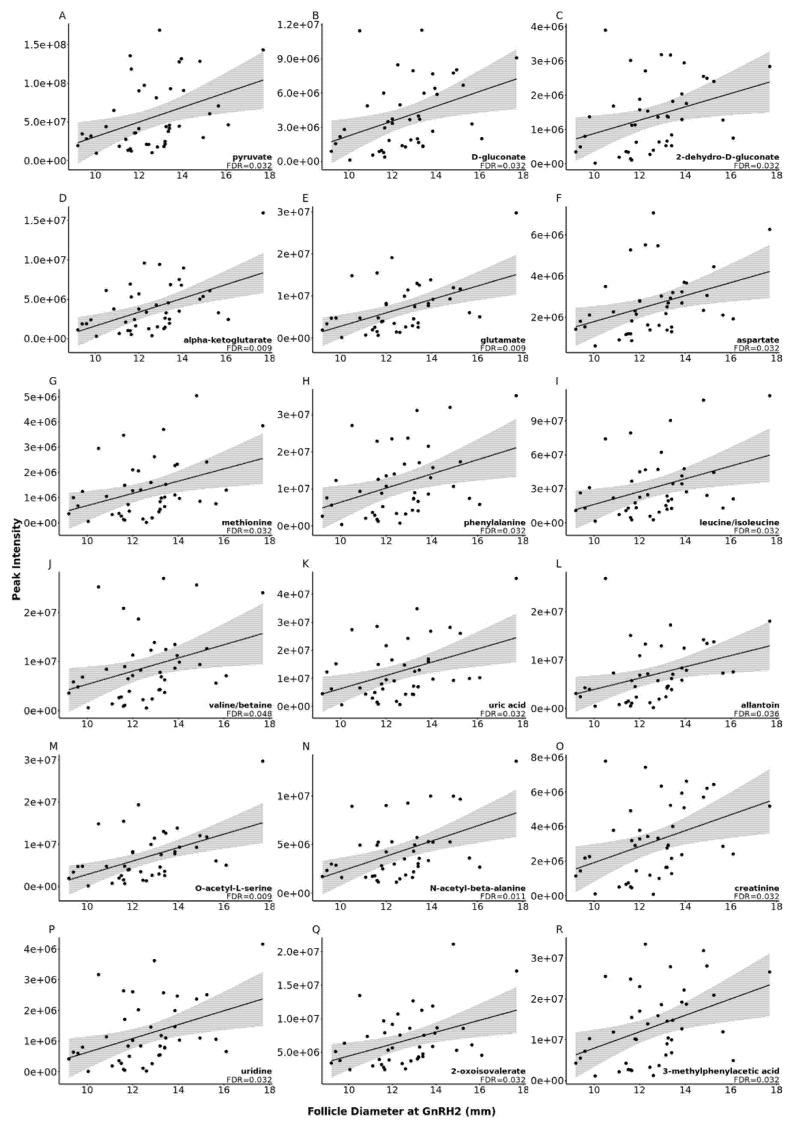
Scatter plots of the correlation between metabolite peak area and follicle diameter at the time of gonadotropin releasing hormone (GnRH2) administration to induce the pre-ovulatory gonadotropin surge for 18 metabolites, depicted in panels (**A**–**R**), that were significantly correlated with increasing follicle diameter at GnRH2 (FDR < 0.05).

Levels of eighteen metabolites were impacted by pre-ovulatory follicle diameter at GnRH2 administration. To this end, as follicle size at the time of GnRH2 administration increased, so did the concentration of each of the 18 metabolites (false discovery rate (FDR) < 0.05; Figure 3). 

Pathway analysis of metabolites significantly correlated with follicle diameter at GnRH2 identified the enrichment of multiple Kyoto Encyclopedia of Genes and Genomes (KEGG) pathways including ‘alanine, aspartate and glutamate metabolism’ (FDR = 0.009), ‘arginine biosynthesis’ (FDR = 0.011), ‘aminoacyl-tRNA biosynthesis’ (FDR = 0.019), and ‘d-glutamine and d-glutamate metabolism’ (FDR = 0.020; Table 1). Significantly enriched pathways and literature review of all metabolites whose abundance was correlated with pre-ovulatory follicle diameter highlighted key processes related to metabolism, transcription, and translation. 

Follicular fluid pyruvate concentration significantly increased with increasing follicle diameter at GnRH2 administration (FDR = 0.032; Figure 3A). The intrafollicular cumulus cells that envelop the oocyte have a high capacity to convert glucose to pyruvate via glycolysis, and glucose consumption by cumulus cells increases during the first 18 h of oocyte maturation [63,64,65,66]. We recently discovered that cumulus cells from small pre-ovulatory follicles had reduced transcript abundance for hexokinase and phosphofructokinase enzymes, which suggests decreased glycolytic capacity for pyruvate production in the cumulus cells of smaller pre-ovulatory follicles [8]. Cumulus cell-produced pyruvate was effectively transferred to both the enclosed oocyte and surrounding medium during in vitro studies, with increased pyruvate concentration detected in the medium as maturation progressed [67]. Therefore, it is logical that increased concentration of follicular fluid pyruvate in the current study may be the result of increased pyruvate production by the cumulus cells of larger follicles. The oocyte has a poor capacity to metabolize glucose and relies on pyruvate transferred from the cumulus cells and surrounding follicular environment to drive oxidative phosphorylation and ATP production for energy demanding processes of maturation, fertilization, cleavage, and early embryonic development [13,14,68].

Interestingly, d-Gluconate and 2-Dehydro-d-gluconate concentration were increased in the follicular fluid of larger follicles (FDR < 0.05; Figure 3B,C, respectively). Gluconate is an alternate carbon source for pyruvate production that can also be converted to 2-Dehydro-d-gluconate. Increased concentrations of d-Gluconate and 2-Dehydro-d-gluconate suggest reduced utilization of gluconate for cumulus cell metabolism in larger pre-ovulatory follicles. 

The KEGG pathways‘ d-glutamine and d-glutamate metabolism’ and ‘alanine, aspartate, and glutamate metabolism’ were significantly enriched with follicular fluid metabolites whose abundance increased with increasing pre-ovulatory follicle size (FDR < 0.03; Table 1). Pathway metabolites alpha-ketoglutarate, glutamate, and aspartate concentrations were increased in the follicular fluid of larger pre-ovulatory follicles (FDR < 0.04; Figure 3D–F, respectively). While the proportion of follicular fluid aspartate concentration compared to total amino acid concentration was higher in 6–8 mm bovine follicles that encased a cumulus-oocyte complex of improved morphology grade, there was no relationship between follicular fluid aspartate concentration and successful cleavage or development to the blastocyst stage [29]. In humans, however, increased follicular fluid concentration of d-aspartate was associated with improved oocyte morphology and increased fertilization rates [69]. Previous studies in bovines have demonstrated the importance of follicular fluid glutamine and glutamate for oocyte energy production and developmental competence. Bovine cumulus-oocyte complexes possess the ability to metabolize glutamine by the enzyme glutaminase [70], and we detected mRNA expression for glutaminase in bovine cumulus cells collected from pre-ovulatory follicles of similar stage and size to the current study [8]. In bovines, when oocytes collected from follicles 6–8 mm in size were submitted to in vitro embryo production, cumulus-oocyte complex morphological quality grade improved as the proportion of follicular fluid glutamate increased. Additionally, follicular fluid glutamate levels were higher when fertilized oocytes successfully cleaved and developed to the blastocyst stage versus when they failed to cleave [29].

The addition of glutamine, pyruvate, and glucose to maturation media increased oocyte nuclear maturation in bovines. Compared to M16 salts media, the addition of glutamine to media significantly increased the percentage of cumulus-enclosed oocytes reaching MII, whereas the addition of glucose, pyruvate, or lactate reduced the percentage of cumulus-enclosed oocytes remaining in the germinal vesicle stage and increased the percentage progressing to MI after 21 h of culture [70]. Interestingly, the metabolism of glucose and glutamine was impacted by the maturation timepoint during in vitro bovine oocyte maturation, with glucose and glutamine metabolism in cumulus-oocyte complexes at their highest at 18 h of maturation [65]. This timepoint is similar to the stage at which samples were collected for the current study and highlights the importance of glucose and glutamine metabolism for energy production during the latter stages of oocyte nuclear maturation.

Increased concentration of alpha-ketoglutarate, glutamate, and aspartate in follicular fluid of larger follicles may be due to increased metabolic activity of the TCA cycle and active transport of these metabolites to the oocyte or follicular fluid. Alpha-ketoglutarate and oxaloacetate are TCA cycle intermediates that can be interconverted with amino acids glutamate and aspartate, respectively [71,72]. Cumulus cells actively transfer glutamate to the oocyte [73], and increased levels of cumulus cell derived alpha-ketoglutarate, glutamate, and aspartate from the TCA cycle could realistically also be transported to the surrounding follicular fluid. 

**Figure 4:**
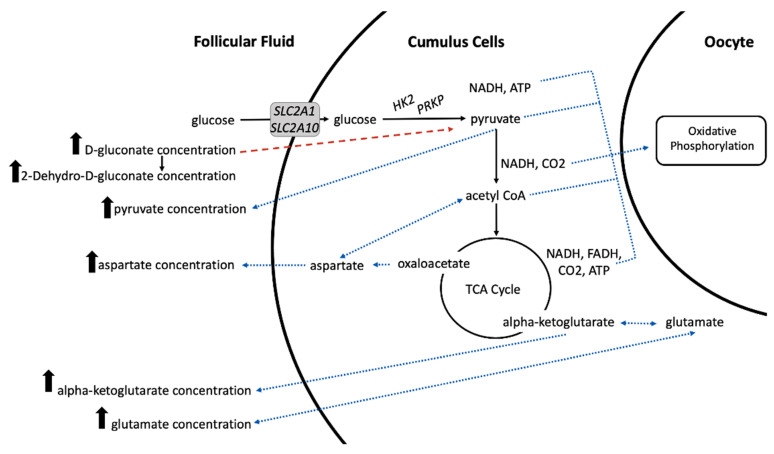
Proposed model of altered glucose metabolism in cumulus cells of small pre-ovulatory follicles. Increased concentration of pyruvate, aspartate, alpha-ketoglutarate, and d-glutamate in the follicular fluid of larger pre-ovulatory follicles is likely the result of increased glucose consumption by the cumulus cells and more efficient glucose, pyruvate, and tricarboxylic acid (TCA) cycle metabolism in the cumulus cells of larger follicles (represented by solid black arrows). Our previous results of increased transcript abundance for glucose transporters (*SLC2A1, SLC2A10*) and glycolytic enzymes (*HK2, PRKP*) in cumulus cells of large versus small pre-ovulatory follicles support the proposed model. Increased production of pyruvate and downstream metabolic products allows increased metabolite abundances to be transferred back into the follicular fluid and to the oocyte to support energy production needed for oocyte maturation, fertilization, cleavage, and early embryo development (represented by blue dotted arrows). Increased concentration of d-gluconate and its downstream product 2-Dehydro-D-gluconate in the follicular fluid of larger follicles is likely due to reduced import of D-gluconate into the cumulus cells (represented by a dashed red arrow) because of preferential import of glucose.

As pyruvate is produced, converted to acetyl CoA, and the TCA cycle is utilized, ATP, NADH, and FADH2 are produced. These metabolic products are critical for oxidative phosphorylation, which is the primary energetic pathway in the oocyte [74]. Increased concentration of pyruvate, d-gluconate, alpha-ketoglutarate, glutamate, and aspartate in the follicular fluid of larger pre-ovulatory follicles lead us to conclude that aberrant glucose metabolism likely exists in the cumulus cells of smaller pre-ovulatory follicles (Figure 4). Reduced glucose metabolism in smaller follicles would lead to a reduction of energetically important stores of ATP, pyruvate, or metabolic intermediates in the oocyte. Decreased metabolic capacity in the cumulus cells of smaller pre-ovulatory follicles would support previous observations of reduced probability of recovering a high quality or cleaved embryo (reported as fertilization success) when cattle are induced to ovulate a smaller dominant follicle <12.5 mm [7].

The KEGG pathway ‘arginine biosynthesis’ was also enriched with follicular fluid metabolites whose abundance increased as follicle diameter at GnRH2 administration increased (FDR = 0.011; Table 1). Increased concentrations of alpha-ketoglutarate, glutamate, and aspartate are consistent with increased glucose metabolism and production of the TCA cycle intermediates or their derivatives (discussed above). Glutamate and aspartate also eventually promote arginine biosynthesis by entering the urea cycle and undergoing conversions that produce the amino acid arginine. Arginine itself was detected in low levels in pre-ovulatory follicular fluid samples of the current study, and arginine concentration was not influenced by follicle diameter (Figure 2; FDR = 0.12). Therefore, we do not hypothesize a biologically relevant impact of enrichment of the ‘arginine biosynthesis’ pathway beyond its relationship to the TCA cycle and improved metabolism in the cumulus cells of larger pre-ovulatory follicles.

The importance of follicular fluid amino acids for oocyte developmental competency expands beyond metabolic support. Amino acids are also important for proteinogenesis, and during maturation, oocytes and cumulus cells undergo morphological changes that involve increased protein synthesis [75]. Enrichment of amino acids in the pathway ‘aminoacyl-tRNA biosynthesis’ (FDR = 0.019; Table 1) indicates that these amino acids are more readily available to be incorporated into protein. Within the follicle and cumulus-oocyte-complex, there are many important signaling molecules, receptors, and enzymes that are made up of amino acids [76]. Deficits in the availability of key amino acids could impact the formation of these molecules and subsequently alter signaling and metabolic pathways as well as developmental events. Our results show an increase in the essential amino acids methionine, phenylalanine, and leucine/isoleucine (FDR = 0.032; Figure 3G–I, respectively) and the nonessential amino acids glutamate and aspartate (FDR < 0.04; Figure 3E,F, respectively) as well as a potential increase in the essential amino acid valine (FDR = 0.048; Figure 3J) as follicle diameter increases. In the bovine, increased total amino acid concentration within the follicular fluid of 6–8 mm follicles was associated with increased oocyte cleavage and development to the blastocyst stage [29]. In vitro, supplementation of cumulus-oocyte-complexes with amino acids supports the acquisition of oocyte developmental competence, and cumulus-oocyte complex utilization and/or excretion of amino acids has been demonstrated to increase during maturation [67]. We hypothesize that increased availability of amino acids in the follicular fluid of larger follicles leads to greater capacity for proteinogenesis and improved developmental competence. 

On the contrary, an in vitro study of bovine cumulus-oocyte complexes collected from 6–8 mm follicles demonstrated that increased proportions of the follicular fluid amino acids histidine, leucine, isoleucine, lysine, methionine, phenylalanine, proline, tyrosine, and valine was negatively associated with cumulus-oocyte-complex morphological assessment and/or oocyte competency for cleavage and/or blastocyst development [29]. In the current study, we detected the presence of methionine, phenylalanine, leucine/isoleucine, and valine/betaine in pre-ovulatory follicular fluid (Figure 2). There was, however, a significant, positive relationship between follicle diameter at GnRH2 administration and the concentration of each amino acid (FDR < 0.05; Figure 3G–J, respectively). Though we did not measure oocyte developmental competency in the current study, oocytes from pre-ovulatory follicles ≥12.5 mm were previously determined to exhibit increased capacity for cleavage and the formation of a high quality embryo [7]. We cannot fully explain the discrepancy between our results and that of the in vitro study, but the variation of follicular fluid source and follicle size/stage of development between the two experiments were likely contributors. 

Not only do amino acids make up the peptide subunits that are used to form proteins, but they also play a role in DNA methylation and initiation of mRNA synthesis. During oocyte development, it is acquiring stockpiles of RNA transcripts and changes in DNA methylation regulate which genes are being actively transcribed. Additionally, during maturation, the chromatin within the oocyte condenses to facilitate the resumption of meiosis [77]. Methionine plays a key role in DNA methylation by forming S-adenosyl methionine (SAM) [77,78]. This molecule plays a key role in the methylation of CpG islands [77]. Multiple dietary studies have shown that increased methionine intake results in increased SAM and hypermethylation [78]. The increased methionine present in the follicular fluid of larger follicles (FDR = 0.032; Figure 3G) could be indicative of increased DNA methylation within the oocytes of larger follicles. 

In addition to its contributions to metabolism and proteinogenesis of follicular cells, the follicular fluid also provides an oxidatively balanced environment for the somatic follicular cells and oocyte. Alpha-ketoglutarate acts as an antioxidant agent, and uric acid is a scavenger of free radicals that is primarily oxidized to allantoin [71,79]. Alpha-ketoglutarate, uric acid, and allantoin were significantly increased in the follicular fluid of larger pre-ovulatory follicles (FDR < 0.04; Figure 3D,K,L, respectively). Such results suggest increased capacity for reduction-oxidation reactions in the follicular fluid, however we cannot speculate if such phenomena lead to improved oocyte developmental competence in larger follicles. Increased concentration of uric acid in the follicular fluid has been previously associated with increasing body mass index in women and poor cumulus-oocyte complex morphology in buffalo cows, indicating that elevated levels of uric acid may be the result of oxidative stress [80,81]. However, the follicular fluid of obese women also possessed higher levels of hormones related to glucose metabolism, and increased abundance of uric acid could be a combative response to potential oxidative stress due to increased metabolic activity of the follicular cells. 

## 3. Materials and Methods

### 3.1. Animal Handling and Synchronization of Pre-Ovulatory Follicle Development

All protocols and procedures were approved by the University of Tennessee Institutional Animal Care and Use Committee (IACUC approved protocol number 2736-1219).

**Figure 5:**
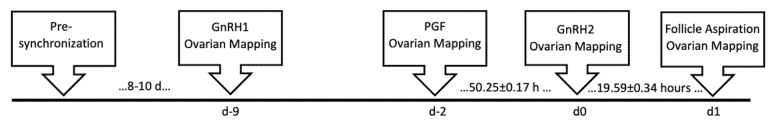
Timeline for synchronization of pre-ovulatory follicle development. GnRH1, gonadotropin releasing hormone administration to turn over a new follicular wave at the onset of synchronization; PGF, prostaglandin F2α; GnRH2, gonadotropin releasing hormone administration to induce the pre-ovulatory gonadotropin surge.

Development of a pre-ovulatory follicle was synchronized in postpartum, suckled beef cattle (Angus; *n* = 130) according to procedures outlined in Figure 5. Estrous cycles were pre-synchronized by the administration of gonadotropin-releasing hormone (GnRH; i.m.; 100 μg; Cystorelin; Boehringer Ingelheim; Ingelheim am Rhein, Germany) and placement of a controlled internal drug release (CIDR; intravaginal; 1.38 g progesterone; Eazi-Breed CIDR^®^; Zoetis Animal Health, Kalamazoo, MI, USA). After seven days, the CIDR was removed and cows were administered prostaglandin F2α (PGF; i.m.; 25 mg; Lutalyse ^®^ HighCon; Zoetis Animal Health, Kalamazoo, MI, USA). Approximately 66 h later, cows were administered a second dosage of GnRH (i.m.; 100 μg; Cystorelin). Cows were then divided into three groups to facilitate transvaginal aspiration with 42–44 cows per group. 

Eight to ten days after pre-synchronization, cows were administered GnRH (GnRH1; i.m.; 100 μg; Cystorelin) on day −9 to start a new follicular wave. On day −2, PGF (i.m.; 25 mg Lutalyse ^®^ HighCon) was administered to lyse corpora lutea. On day 0, cows received a second dosage of GnRH (GnRH2; i.m.; 100 μg; Cystorelin) to induce a pre-ovulatory gonadotropin surge.

Estrous detection patches (Estrotect^®^; Rockway Inc; Spring Valley, WI, USA) were placed on all cows at the time of PGF administration during pre-synchronization and on day −9. Patches were visually assessed 66 h after pre-synchronization PGF administration to determine estrous expression during pre-synchronization and on days −2 and 0 of synchronization to detect any animals that displayed estrus between GnRH1 and PGF (removed from study because of failed synchronization, *n* = 0) or between PGF and GnRH2 (endogenous gonadotropin surge, not included in study, *n* = 2). Patches were scored on a scale of 0–4 with 0 being missing, and 1–4 equating to <25% rubbed (patch = 1), 25–50% rubbed (patch = 2), 50–75% rubbed (patch = 3), and >75% rubbed (patch = 4). Estrous expression was recorded if patch score equaled 3 or 4. 

On days −9, −2, 0, and 1, ovaries of all cattle were examined by an experienced technician using trans-rectal ultrasonography with a Samsung HM70A ultrasound and CF4-9 convex probe. All follicles > 7 mm in diameter and all corpora lutea were recorded. Follicle size was calculated for all recorded follicles by averaging the measures of the largest diameter and the diameter perpendicular to it. Body weights of all animals were collected and body condition score ([82] scale of 1, emaciated-9, obese) was assigned on day −9.

### 3.2. Transvaginal Aspiration for Collection of Follicular Fluid from the Pre-Ovulatory Follicle

On day 1, approximately 20 h after GnRH2 administration, each cow’s largest follicle underwent transvaginal aspiration by 1 of 4 experienced technicians to collect the pre-ovulatory follicle contents. Prior to aspiration, all cows received a spinal block via administration of lidocaine (2% lidocaine, 5 mL) into the spinal cord at the first intercoccygeal space of the tailhead. The perineal area of each cow was then cleaned of all contaminants and an ultrasound guided aspiration device attached to a Samsung HM70A ultrasound and CF4-9 convex probe was inserted into the anterior vagina. The ultrasound device consisted of an 18-gauge needle and a series of tubing to facilitate removal of follicular contents. The ovary containing the pre-ovulatory follicle was located and positioned for follicle aspiration before the needle was gently pushed through the vaginal wall and guided through the ovarian cortex into the antrum of the pre-ovulatory follicle. Follicular fluid was withdrawn into a clean 12 mL syringe before the syringe was removed, replaced, and the follicle lavaged with PVA TL-HEPES media multiple times to collect remaining follicular cells.

### 3.3. Follicular Fluid Processing

Follicular fluid was deposited into a 4-well petri plate and searched to find the cumulus-oocyte-complex which was removed and snap frozen for a subsequent study. The follicular fluid was then collected into 1.7 mL tubes and centrifuged at 4 °C for 5 min at 500× *g* to remove the remaining cellular debris. The follicular fluid supernatant was distributed amongst 2 mL cryovials and snap frozen in liquid nitrogen for storage at −80 °C until further processing. 

### 3.4. UHPLC-HRMS Metabolomics

Forty-three follicular fluid samples were selected for metabolomics processing. Follicular fluid samples were thawed on ice, and 60µL aliquots of each sample were placed into individual 2 mL tubes. Each sample was analyzed by ultra-high performance liquid chromatography—high resolution mass spectrometry (UHPLC-HRMS) at the University of Tennessee Biological and Small Molecule Mass Spectrometry Core (RRID: SCR_021368). Briefly, metabolites were extracted from the follicular fluid using a 20:40:40 water/methanol/acetonitrile solution with 0.1 M formic acid [83,84]. The metabolomes of each sample were separated on a Synergy Hydro RP, 2.5 μm, 100 mm × 2.0 mm column (Phenomenex, Torrance, CA, USA) at 25 °C. The solvents for the elution were: phase A: 97:3 methanol/water with 11 mM tributylamine and 15 mM acetic acid and phase B: 100% methanol. The solvent gradient from 0 to 5 min was 100% A: 0% B, from 5 to 13 min was 80% A: 20% B, from 13 to 15.5 min was 45% A: 55% B, from 15.5 to19 min was 5% A: 95% B, and from 19 to 25 min was 100% A: 0% B with a flow rate of 200 μL/min. Detection of the metabolome components was accomplished using an Exactive Plus Orbitrap mass spectrometer (Thermo Fisher Scientific, Waltham, MA, USA) fitted with an electrospray ionization (ESI) probe operated in negative mode. The scan range was 72–1000 *m/z*, the resolution was set to 140,000, and the acquisition gain control target to 3 × 10^6^.

### 3.5. Primary Data Analysis

Files were generated by the HRMS in the Xcalibur (RAW) format and were converted to the open-source mzML format [85] via the open-source msconvert software, which is part of the ProteoWizard package [86]. MAVEN (mzroll) software, Princeton University [87,88], which uses a grouping algorithm for non-linear retention time alignment, was used to pick peaks, integrate intensities, and visualize the data and extracted ion chromatograms. Pre-processed data from MAVEN was used to conduct all further biological and statistical analyses.

### 3.6. Statistical Analyses

All analytical procedures were performed using R software version 3.6.3 [89], and the corresponding code is available online (https://github.com/CaseyRead/Read_etal_2021_metabolome; accessed on 31 August 2021). Analysis of variance was used to determine any effects of cow age and BCS on pre-ovulatory follicle diameter at GnRH2 administration, and a linear model was used to determine any effects of cow weight, days postpartum at aspiration, days between PGF and GnRH2, and hours between GnRH2 and aspiration on pre-ovulatory follicle diameter at GnRH2.

A heatmap was constructed to visualize differences in metabolite peak area among metabolites and follicle diameters. Data for each metabolite was tested for normality by performing a Shapiro Wilk test and by plotting the residuals for visual evaluation of normality. Each metabolite was determined to have an approximately normal distribution. A linear model was used to determine the relationship between pre-ovulatory follicle size and follicular fluid metabolite peak area values in the 38 metabolites identified in our samples. Metabolites were considered significantly correlated to pre-ovulatory follicle diameter at GnRH2 administration if FDR < 0.05 was observed. Metaboanalyst 5.0 [90] was used to perform KEGG pathway enrichment analysis of the 18 metabolites that were significantly correlated to follicle diameter at GnRH2. Enrichment of pathways was determined to be significant if the FDR was <0.05. 

## 4. Conclusions

In conclusion, we identified a total of 38 metabolites in the follicular fluid of bovine pre-ovulatory follicles. Eighteen metabolites were positively correlated to increasing follicle diameter at the time of GnRH administration to induce the pre-ovulatory gonadotropin surge. Pathway and individual analysis of these metabolites revealed that pathways and substrates involved in glucose metabolism, energy production, and proteinogenesis were present in higher levels in the follicular fluid of larger follicles. The follicular fluid microenvironment plays a key role in oocyte acquisition of developmental competence by providing the cumulus-oocyte complex with nutrients and metabolic substrates. Decreased availability of metabolites and proteinogenic components to cumulus-oocyte-complexes from smaller follicles likely contributes to the reduced oocyte developmental competence and lower pregnancy rates observed when beef cows are induced to ovulate a small pre-ovulatory follicle. Future studies that include assessment of oocyte developmental competency and incorporate metabolic parameters in the follicular fluid and cumulus cells of oocytes resulting in high or low rates of successful embryo cleavage and/or blastocyst development will strengthen the current conclusions.

## Figures and Tables

**Table 1:** KEGG pathways significantly enriched with metabolites that were significantly correlated with increasing pre-ovulatory follicle diameter.

Pathway	Pathway Name	Match Status *	FDR	Differentially Abundant Metabolites in Pathway ^†^
bta00250	alanine, aspartate and glutamate metabolism	4/28	0.009	C00026 (Alpha-Ketoglutarate) C00049 (Aspartate) C00025 (Glutamate) C00022 (Pyruvate)
bta00220	arginine biosynthesis	3/14	0.011	C00026 (Alpha-Ketoglutarate) C00049 (Aspartate) C00025 (Glutamate)
bta00970	aminoacyl-tRNA biosynthesis	4/48	0.019	C00049 (Aspartate) C00025 (Glutamate) C00073 (Methionine) C00079 (Phenylalanine)
bta00471	d-glutamine and d-glutamate metabolism	2/5	0.020	C00026 (Alpha-Ketoglutarate) C00025 (Glutamate)

* number of differentially abundant metabolites in pathway/total number of metabolites in pathway; ^†^ displayed as KEGG identifier number (Name); KEGG, Kyoto Encyclopedia of Genes and Genomes; FDR, false discovery rate.

## Data Availability

The data presented in this study are available on request from the corresponding author. The data are not publicly available due to their inclusion in a larger study that was not yet published at the time of this manuscript’s submission.
